# Intradiscal injection of human recombinant BMP-4 does not reverse intervertebral disc degeneration induced by nuclectomy in sheep

**DOI:** 10.1016/j.jot.2022.08.006

**Published:** 2022-09-23

**Authors:** Jie Du, João P. Garcia, Frances C. Bach, Anna R. Tellegen, Sibylle Grad, Zhen Li, René M. Castelein, Björn P. Meij, Marianna A. Tryfonidou, Laura B. Creemers

**Affiliations:** a Department of Orthopedics, University Medical Center Utrecht, Utrecht, the Netherlands; b Department of Clinical Sciences, Faculty of Veterinary Medicine, Utrecht University, the Netherlands; c AO Research Institute Davos, Davos, Switzerland

**Keywords:** BMP-4, Intervertebral disc regeneration, Nucleus pulposus, Annulus fibrosus, Subchondral bone remodeling, Bone formation, AB/PR, Alcian blue and picrosirius red, ADAMTS, A disintegrin and metalloproteinase with thrombospondin motifs, AF, Annulus fibrosus, BMPs, Bone morphogenetic proteins, CLBP, Chronic low back pain, DHI, Disc height index, ECM, Extracellular matrix, EP, Endplate, GAG, Glycosaminoglycan, GDF-8, Growth and differentiation factor-8, H&E, Hematoxylin and eosin, IVD, Intervertebral disc, IVDD, Intervertebral disc degeneration, Ki-67, Nuclear Ki-67 protein, LBP, Low back pain, Micro-CT, Micro-computed tomography, MMPs, Matrix metalloproteinases, MRI, Magnetic resonance imaging, NP, Nucleus pulposus, RPL19, Ribosomal protein L19, Saf-O FG, Safranin-O and fast green, SOX-9, SRY-box transcription factor-9, TGF-β, Transforming growth factor-beta, TRAP, Tartrate-resistant acid phosphatase

## Abstract

**Background:**

Intervertebral disc (IVD) degeneration is suggested as a major cause of chronic low back pain (LBP). Intradiscal delivery of growth factors has been proposed as a promising strategy for IVD repair and regeneration. Previously, BMP-4 was shown to be more potent in promoting extracellular matrix (ECM) production than other BMPs and TGF-β in human nucleus pulposus (NP) cells, suggesting its applicability for disc regeneration.

**Methods:**

The effects of BMP-4 on ECM deposition and cell proliferation were assessed in sheep NP and annulus fibrosus (AF) cells in a pellet culture model. Further, a nuclectomy induced sheep lumbar IVD degeneration model was used to evaluate the safety and effects of intradiscal BMP-4 injection on IVD regeneration. Outcomes were assessed by magnetic resonance imaging, micro-computed tomography, histological and biochemical measurements.

**Results:**

In vitro, BMP-4 significantly increased the production of proteoglycan and deposition of collagen type II and proliferation of NP and AF cells. Collagen type I deposition was not affected in NP cells, while in AF cells it was high at low BMP-4 concentrations, and decreased with increasing concentration of BMP-4. Intradiscal injection of BMP-4 induced extradiscal new bone formation and Schmorl's node-like changes in vivo. No regeneration in the NP nor AF was observed.

**Conclusion:**

Our study demonstrated that although BMP-4 showed promising regenerative effects in vitro, similar effects were not observed in a large IVD degeneration animal model.

**The Translational Potential of This Article:**

The contradictory results of using BMP-4 on IVD regeneration between *in vitro* and *in vivo* demonstrate that direct BMP-4 injection for disc degeneration-associated human chronic low back pain should not be undertaken. In addition, our results may also shed light on the mechanisms behind pathological endplate changes in human patients as a possible target for therapy.

## 1 Introduction

Low back pain (LBP) is one of the most common musculoskeletal disorders and a leading cause of disability worldwide [1]. LBP impairs patient quality of life, and particularly chronic LBP (CLBP) imposes a heavy burden on economy, healthcare and social systems. Intervertebral disc (IVD) degeneration (IVDD) is widely recognized as a major cause of CLBP [[2], [3], [4]]. IVDD progresses with age and is associated with mechanical stress, trauma, infection, genetic predisposition, and lifestyle [5]. Additionally, the spontaneous regeneration of the IVD is very limited, which is attributed to the low number of resident progenitor cells and the avascular nature of adult IVDs [6].

IVDD is characterized by abnormal extracellular matrix (ECM) metabolism, with decreasing anabolism and increasing catabolism. In degenerative IVDs, the proteoglycan content is reduced together with changes in collagen production, where collagen type II is being degraded and replaced by increasing amounts of collagen type I [7]. This is associated with enhanced activity of catabolic enzymes, such as matrix metalloproteinases (MMPs) and a disintegrin and metalloproteinase with thrombospondin motifs (ADAMTS) [8]. All these factors result in disorganization of ECM architecture, tears and clefts in IVD, and loss of water and IVD height [9]. Recently, the inflammatory environment and neoinnervation have been suggested as a source of the ensuing discogenic back pain [10]. Current therapies for CLBP mainly aim to relieve pain but do not restore the physiological composition and function of the degenerated IVDs. In this context, strategies for the repair and regeneration of IVD are promising alternatives. Approaches using growth factors to induce regeneration have been widely studied, because they effectively induce IVD cell proliferation and ECM production, and they can be potential minimally invasive [11].

Bone morphogenetic proteins (BMPs), a subfamily of the transforming growth factor-beta (TGF-β) superfamily, are widely known as potent inducers of bone formation [12]. Moreover, BMPs have also been studied for their role in chondrogenesis and the maintenance of ECM in both IVD and articular cartilage [13,14]. Importantly, several studies showed that BMPs promote IVD regeneration both in vitro and in vivo. BMP-2 and BMP-7 and their heterodimers have been demonstrated to promote ECM anabolism in nucleus pulposus (NP) and/or annulus fibrosus (AF) cells in different species [[15], [16], [17]]. Intradiscal injection of BMP-7 increased disc height and proteoglycan content in both annular puncture and chondroitinase ABC-induced rabbit disc degeneration models [18,19]. However, these results were not replicated in a canine model of mild IVDD [20]. Rabbit models in general show a higher spontaneous regenerative capacity in IVD than other species, possibly owing to the presence of notochordal cells in their NPs up to adulthood. Therefore, they may not be the optimal model for regenerative interventions of the IVD [21]. In this context, large animal models such as dog and sheep models of IVDD may be a better alternative for studying IVD regeneration, as their IVDs are similar to human in size and cell biology [22].

Previously, we compared BMP-2, BMP-4, BMP-6, BMP-7, and their combinations and heterodimers, for their regenerative effect on the pellet culture model of human NP cells or NP cells co-cultured with bone marrow mesenchymal stromal cells [23]. In this study, BMP-4 was identified as the most potent in inducing glycosaminoglycan production and deposition, suggesting a regenerative effect could be achieved upon direct intradiscal injection [23]. In order to investigate the applicability of BMP-4 for IVDD treatment, the effects of BMP-4 on IVDD were evaluated in the present study in vitro and in a large animal model of IVDD. In vitro, the effects of BMP-4 on ECM deposition and cell proliferation were assessed in both sheep NP and AF cells, because the AF cells could be affected by intradiscal injected BMP-4. In vivo, IVDD was induced by nuclectomy in a sheep model, and BMP-4 was intradiscally injected into the degenerative IVDs to evaluate the safety and effects on IVD regeneration. Outcomes were assessed by magnetic resonance imaging (MRI), micro-computed tomography (Micro-CT), histological and biochemical measurements.

## 2 Materials and methods

### 2.1 Ethics statement

All procedures involving animals were approved and conducted in accordance with the guidelines - and described in the protocol number AVD108002015282 provided by the central national committee for animal experiments and overseen by the Local Welfare Body, as required by Dutch regulation.

### 2.2 Isolation and culture of NP and AF cells

NP and AF cells were isolated from laboratory Swifter sheep's healthy IVDs. Swifter breed is known to include animals heterozygous and homozygous for the allele of the growth and differentiation factor-8 (GDF-8) gene at the 3′ untranslated region +6723. As this may affect the regenerative response [24], animals for in vitro and in vivo experiments were genotyped and only animals homozygous for the gg allele were used; this phenotype excludes aberrant GDF-8 signaling. IVDs were obtained from remnants of other experiments, and macroscopically scored according to Thompson grading, and grade I and II IVDs were included. NP tissue was carefully separated avoiding the transitional zone, and the AF was identified by its clear lamellar structure. Minced tissue was firstly digested with 0.004% DNase (D4138-80KU, Sigma-Aldrich), 0.2% pronase (11459643001, Roche Diagnostics GmbH) for 1 ​h at 37 ​°C, and subsequently digested with 0.004% DNAse and 0.05% collagenase type II (LS004176, Worthington Biochemical) for NP or 0.004% DNAse and 0.1% collagenase type II for AF overnight at 37 ​°C. All the digestion buffers were prepared in an antibiotic plus DMEM medium (DMEM(1 ​× ​) ​+ ​GlutaMAX™ (31966021, Gibco), 2% penicillin/streptomycin (P/S, 15140122, Gibco), 20 ​μg/mL amphotericin-B (15290026, Gibco) and 50 ​μg/mL gentamicin sulfate (BW17-518Z, Lonza™ BioWhittaker™ Antibiotics)). Single cells seeded with antibiotic plus DMEM medium supplemented with 10% fetal bovine serum (FBS) (Biowest, Missouri, USA) at a density of 3000–4500 ​cells/cm^2^ as passage 0. After passage one, cells were cultured in expansion medium, DMEM(1 ​× ​) ​+ ​GlutaMAX™ supplemented with 1% P/S, 10% FBS, and 1 ​ng/mL basic fibroblast growth factor (bFGF, PHP105, AbD Serotec). Both NP and AF cells were cryopreserved at passage 1.

### 2.3 Pellet culture and BMP-4 treatment

NP and AF cells were expanded until passage 2. They were pelleted at 2.5 ​× ​10^5^ ​cells in a round bottom ultra-low attachment 96-well plate (Costar, ME, USA) by centrifugation at 500*g* for 5 ​min. Pellets were cultured in 200 ​μL pellet culture medium (DMEM(1 ​× ​) ​+ ​GlutaMAX™ supplemented with), 2% bovine serum albumin (10735086001, Roche Diagnostics GmbH), 1% insulin-transferrin-selenium ​+ ​premix (ITS+) (354,352 Corning), 20 ​mg/mL proline (P5607, Sigma-Aldrich), 1% P/S, and 20 ​mM ascorbate-2-phosphate (Sigma-Aldrich)) with or without human recombinant BMP-4 (kind gift of dr Loredana Cecchetelli, Rome, Italy) at four different concentrations, 0.04 ​nM (1.36 ​ng/mL), 0.4 ​nM (13.6 ​ng/mL), 2 ​nM (68 ​ng/mL), and 4 ​nM (136 ​ng/mL), the concentration based on previous study [23]. Media were renewed twice a week. NP and AF pellets cultured for 28 days were used for biochemical analysis and histological and immunohistochemical staining. NP pellets cultured for 2 or 7 days were used for gene expression analysis.

### 2.4 Gene expression analysis

NP pellets cultured for 2 or 7 days were collected by adding 1 ​mL TRIzol™ reagent (Invitrogen) per 2 pellets for RNA isolation. Then pellets were minced by pipette tips. Before RNA isolation, samples were mixed with 70% ethanol 1 ​mL. Following RNA isolation was performed by using RNeasy Mini Kit (QIAGEN) according to the manufacturer's protocol. Reverse transcription was performed using High-Capacity cDNA Reverse Transcription Kit (4368814, Applied Biosystems). qRT-PCR was conducted on CFX96 Touch Real-Time PCR Detection System (BIO-RAD) with iTaq Universal SYBR Green Supermix (BIO-RAD). Primers: endogenous control, ribosomal protein L19 (RPL19), forward: 5′- AGCCTGTGACTGTCCATTCC-3′, reverse: 5′-ACGTTACCTTCTCGGGCATT-3′ [25]; nuclear Ki-67 protein (Ki-67) forward: 5′-AAGATTCCAGTCCCCGTTCA-3′, reverse: 5′- TGAGGAACGAACACGACTGG-3′; SRY-box transcription factor-9 (SOX-9) forward: 5′- TTCGTGAAGATGACCGACGA-3′, reverse: 5′- AACTTGTCCTCCTCGCTCTC-3′. Data were analyzed using the 2 ^−ΔΔCT^ method.

### 2.5 Animal experiment design

Four female Swifter sheep, 2 years old, were used in this study. All were confirmed by PCR to be gg wild type for the 3’ untranslated region +6723 of GDF-8. Multisegment modeling was used in the current study and applied by various previous authors to address the 3Rs principle by reducing animal use. The adjacent healthy discs of degenerative positions did not show a degeneration in past studies using this approach in canines and goats [14,20,26]. And no evidence was found for effects of substances injection either [20,26]. Degeneration of lumbar IVDs was induced by nuclectomy, and 6 weeks afterwards, degenerative IVDs were treated by intradiscal injection of BMP-4 (D-BMP4), or by a negative protein control consisting of a random ^19^F peptide (sequence: CF_3_CO–NH-Lys–(CO–CF_3_)-DNRAHLHIDYHTDSD-COOH; D-sham) (This peptide injected IVDs were involved in a study to investigate the retention of peptide in IVD). As control for injection, also healthy IVDs were injected with this peptide (H-sham). Healthy discs without injection served as non-treated control (Healthy) (IVD levels and treatment layout as Supplementary Table 1). Three months after injection, sheep were euthanized, and samples were analyzed.

### 2.6 Sheep IVD nuclectomy

IVDD in the sheep was induced by surgical nuclectomy under anesthesia as previously reported [27]. Briefly a longitudinal incision was made over the lumbar spine from the left side, and blunt dissection between the lumbar muscles was employed to facilitate visualization of the IVDs at L1- L2, L3 - L4, and L5 - L6 of each sheep and localization of the IVD segments was confirmed by fluoroscopy. Incision of the AF was performed with surgical blade no.11, whereafter NP tissue was partially removed with a round 1 ​mm ball-tipped probe. After nuclectomy, the muscle, fascial, subcutaneous and skin incisions were closed separately. Sheep received a single prophylactic antibiotic (amoxicilline & clavulanic acid, 10 ​mg/kg, intravenous, administration prior to surgery and Neopen (0.05 ​mL/kg containing 100 ​mg Neomycin and 200 ​mg Procaine benzylpenicilline per mL) for three days post-surgery. They also received analgesia by Buprenorfine (BuTrans pleister), 5 ​mg, release 5 ​μg/h for 7 days and intravenous and subcutaneous administration Meloxicam 0.5 ​mg/kg at day 0 and day 1–3 respectively after surgery. They were allowed ad libitum activity with free access to food and water.

### 2.7 Intradiscal injection

Sheep were anesthetized and lumbar IVDs were accessed by surgery as reported previously [27]. IVDs were exposed at the right side (opposite to nuclectomy). Twenty-four lumbar IVDs from 4 sheep, were divided into 4 groups (4 discs, Healthy; 8 discs, H-sham; 6 discs, D-sham; 6 discs, D-BMP4) (Supplementary Table 1). A 27 ​G syringe needle was inserted into the NP center, confirmed by fluoroscopy, and 200 ​μg of BMP-4, the injected dose based on a previous large animal study using BMP-7 [20], or a random peptide was injected into IVDs (dissolved in sterilized ultrapure water, 130 ​μL final volume for degenerative IVDs and 65 ​μL for healthy IVDs). Sheep received antibiotics and analgesia similar to the first operation.

### 2.8 Magnetic resonance imaging

MRI of lumbar spine was performed under anesthesia prior to the surgery for intradiscal injection and immediately after euthanasia at three months after injection. MRI scans were obtained by using a Philips Ingenia 1.5 ​T scanner (Philips, Eindhoven, Netherlands). The sheep were positioned in dorsal recumbency with the pelvic limbs extending caudally. The MR protocol included a sagittal and transverse T2-weighted Turbo Spin Echo (time of repetition (TR) ​= ​3000, time of echo (TE) ​= ​110 ​ms, slice thickness ​= ​2.5 ​mm) sequence, a T1-weighted Turbo Spin Echo (TR ​= ​400 ​ms, TE ​= ​8 ​ms, slice thickness ​= ​2.5 ​mm). A sagittal multiple spin-echo T2w sequence for quantitative T2 mapping (using custom script in MeVisLab v3.1, MeVis Medical Solutions AG, Bremen, Germany) with a field of view (FOV) ​= ​75 ​× ​219 ​mm, acquisition matrix ​= ​96 ​× ​273, slice thickness ​= ​3 ​mm, TR ​= ​2000. Thirteen echoes were acquired with TE ​= ​13–104 ​ms with 13 ​ms echo spacing. All images were assessed by a board-certified veterinary radiologist (Enterprise Imaging, version 8.1.2, Mortsel, Belgium). The lumbar discs were graded according to the Pfirrmann grading system on T2-weighted by two observers [28]. The disc height index (DHI) was measured on T2 weighted images obtained prior to injection and at 3 months after injection by using the method of Masuda et al. [29]. Three T2-weighted images (mid-sagittal and the parasagittal directly left and right of the mid-sagittal slice) of each IVD segment were used to measure the DHI, and results were averaged.

### 2.9 Sheep sample collection

Following euthanasia, the lumbar spine was harvested and used to perform micro-CT scans. Then single intact IVDs were obtained with partial vertebrae on both sides. The IVDs were cut into two equal half parts at the middle sagittal plane, and sagittal planes were imaged using a Canon 600D digital camera and EF-S 18–55 ​mm lens (Canon, Tokyo, Japan) at a fixed distance to evaluate the macroscopic degenerative level of each IVD according to the Thompson grading system [30]. The grading was performed in random order by two independent investigators blinded to treatment. One half of each IVD was fixed in 4% neutral phosphate buffered formaldehyde (w/v) for histology, and the other half was snap-frozen in liquid nitrogen and stored at −80 ​°C for biochemical analyses.

### 2.10 Micro-computed tomography

Each IVD was individually scanned with a Micro-CT scanner(Quantum FX, Perkin Elmer, USA) at a voxel size of 143 ​μm^3^ with 90 ​kV tube voltage and 200 ​μA tube current for 120 ​s 3D reconstruction was carried out automatically after completion of each scan using the scanner's software (Quantum FX μCT software, Perkin Elmer, USA). Image analysis was performed using Fiji (software version 1.50, National Institutes of Health, Bethesda, USA). 3D images were used to evaluate new bone formation around IVDs; no osteophyte (0), osteophyte found (1). Sagittal 2D images were used to evaluate the presence of subchondral bone defects; no defect (0), defects found (1).

### 2.11 Biochemistry for glycosaminoglycan (GAG), DNA, and collagen content

GAG measurements were performed in pellet culture media, cell pellets, and IVD tissue. DNA and collagen content were measured in both pellets and IVD tissue.

After culturing for 28 days, three pellets per condition per donor were separately digested in 300 ​μL papain buffer (250 ​μg/mL papain (P3125, Sigma-Aldrich) with 1.57 ​mg/mL cysteine HCl (C7880, Sigma-Aldrich)) overnight at 60 ​°C. These digested solutions were used to measure GAG, collagen, and DNA content.

Frozen IVDs were cut transversely at 30 ​μm thickness with a cryostat microtome (Thermo Fisher, USA). NP and AF tissue were collected separately. They were lysed in cOmplete lysis-M EDTA-free buffer (Roche Diagnostics GmbH, Mannheim, Germany), 1 ​mL for NP and 1.5 ​mL for AF, on a rotor at 4 ​°C overnight (Because the tissue lysate is needed to investigate ^19^F labelled peptide retention in IVDs). Tissue lysates of NP and AF, 500 ​μl, were collected after centrifuging at 10,000 ​g at 4 ​°C for 20 ​min. The remaining NP and AF tissue were freeze-dried and weighed. The tissue lysates and freeze-dried tissue were digested in papain buffer (lysate: papain, v: v ​= ​1: 10 for the lysate, 1 ​mg dry weight per 150 ​μL papain buffer for the freeze-dried tissue) at 60 ​°C overnight. Papain-digested samples were used to measure GAG, collagen, and DNA content in AF and NP tissue. The total content of GAG, collagen, and DNA was normalized to the weight of freeze-dried tissue of NP and AF separately.

The 1,9-dimethyl-methylene blue (DMMB, Sigma-Aldrich) assay was used to quantify GAG content using chondroitin sulfate (C4384, Sigma-Aldrich) as a standard, and the absorbance was read at 540/595 ​nm with a VersaMax microplate reader (Molecular Devices, San Jose, CA, USA).

The hydroxyproline assay was used to measure total collagen content. Papain-digested samples were freeze-dried overnight, and hydrolyzed in 4 ​M NaOH at 108 ​°C overnight, then neutralized by 1.4 ​M citric acid (Fluka, Switzerland). Samples were incubated with Chloramine T reagent (Merck, Germany) for 20 ​min, then incubated with dimethylaminobenzaldehyde reagent (Merck, Germany) at 60 ​°C for 20 ​min. The absorbance was read at 570 ​nm by VersaMax microplate reader. The concentrations were calculated by using hydroxyproline (Merck, Germany) as a standard.

The Quant-iT™ PicoGreen™ dsDNA Assay Kit (Invitrogen, USA) was used to measure DNA content according to the manufacturer's protocol, using λDNA as standard. Fluorescence was read in a POLARstar Optima fluorescence microplate reader (Isogen Life Science, Utrecht, The Netherlands) at 485 ​nm excitation and 530 ​nm emission.

### 2.12 Histological and immunohistochemical staining

Two pellets per condition per donor were fixed overnight in 4% neutral phosphate buffered formaldehyde (w/v) and dehydrated and embedded in paraffin. Sections of 5 ​μm thickness were used for histological and immunohistochemical staining.

Fixed half IVDs were decalcified in 0.5 ​M EDTA dissolved in distilled water for 4 months, with two changes weekly and re-fixed for 2 days every 2 weeks. Full decalcification was verified by micro-CT. Decalcified IVDs were dehydrated and embedded in paraffin. Sagittal sections of 5 ​μm thickness were used for histological and immunohistochemical staining.

Hematoxylin and eosin (H&E) staining was performed with Mayer's hematoxylin solution (Merck, Germany) and then 2% eosin (Merck, Germany).

Safranin-O/Fast green (Saf-O FG) staining was performed by staining with Weigert's Hematoxylin (640,500, Clin-Tech, UK), 0.125% Safranin-O (58,884, Sigma-Aldrich), and finally 0.4% Fast Green (F7252, Sigma-Aldrich).

Alcian Blue/Picrosirius Red (AB/PR) staining was performed, by first staining with Weigert's hematoxylin (Clin-Tech, UK), followed by 1% Alcian Blue (05500, Fluka, Switzerland) at pH 2.5, and then 0.1% Picrosirius red (80,115, Klinipath, Belgium). The IVD histological grading was performed in random order and by two independent investigators blinded for treatment under Olympus BX41 microscope based on H&E, AB/PR, and Saf-O FG staining and according to a previously described grading system [22]. Eight parameters were included, NP cell loss and cell death, NP cell clusters, NP matrix staining, AF morphology, AF cellular and matrix metaplasia/distinction between AF and NP, tears and cleft formation in AF/NP, endplate (EP) morphology, and bone modeling at the external AF/bone interface.

Tartrate-resistant acid phosphatase (TRAP) staining was performed to detect osteoclasts in the subchondral bone. Sections were pre-incubated with 0.2 ​M acetate buffer-tartaric acid for 20 ​min at 37 ​°C. Subsequently, sections were incubated in 0.5 ​mg/mL naphthol AS-MX phosphate (Sigma-Aldrich) and 1.1 ​mg/mL Fast red TR salt (Sigma-Aldrich) for another 3 ​h. Sections were counterstained with Mayer's hematoxylin. Osteoclasts were defined as multinucleated TRAP-positive cells and counted in the subchondral bone along whole two sides of IVD, in an area defined from the bottom edge (cartilaginous endplates) to the up edge (vertebrae) of view under a 20-times objective lens of Olympus BX41 microscope. The count was performed in a random order blinded for treatment.

Immunohistochemistry staining of collagen type I and II was performed after blocking endogenous peroxidase activity with 0.03% hydrogen peroxidase, antigen retrieval with 1 ​mg/mL pronase followed by 1 ​mg/mL hyaluronidase (H3506, Sigma-Aldrich) and blocking with PBS ​+ ​5% bovine serum albumin (BSA, 10735078001, Roche Diagnostics GmbH). Primary antibodies for collagen type I (2 ​μg/mL, rabbit, EPR7785, Abcam, Cambridge, UK), type II collagen (0.4 ​μg/mL, mouse, DSHB II-II6B3, DSHB, IA, USA) or isotype control (DAKO, Glostrup, Denmark) (host species and concentrations matched with the primary antibody) were diluted in PBS ​+ ​5% BSA and incubated with sections overnight at 4 ​°C. Species-specific HRP-secondary antibodies (Immunologic a WellMed Company, Duiven, The Netherlands) were incubated with the sections for 1 ​h at room temperature. Then staining was performed using the liquid DAB ​+ ​Substrate Chromogen System (DAKO, Glostrup, Denmark). Sections were counterstained with Mayer's hematoxylin solution, dehydrated and mounted. Images were taken with an Olympus BX51 upright microscope with an Olympus SC50 camera (Olympus, Tokyo, Japan).

### 2.13 Statistical analyses

Statistical analyses were performed using the IBM SPSS statistics software, version 20. As the data were not normally distributed which was defined by Shapiro-wilk normality test. Mann-Whitney U test was used to determine differences between two groups; Kruskal Wallis with post-hoc test was used to determine differences among more than two groups. P ​< ​0.05 was considered statistically significant.

## 3 Results

### 3.1 Effects of BMP-4 on sheep NP and AF cells in vitro

To verify that sheep IVD cells responded similarly to BMP-4 as human IVD cells [23], sheep NP and AF cells were cultured in pellets to allow for tissue formation.

#### 3.1.1 BMP-4 promoted tissue formation, viability and cell content in NP and AF cell pellet culture

**Fig. 1:**
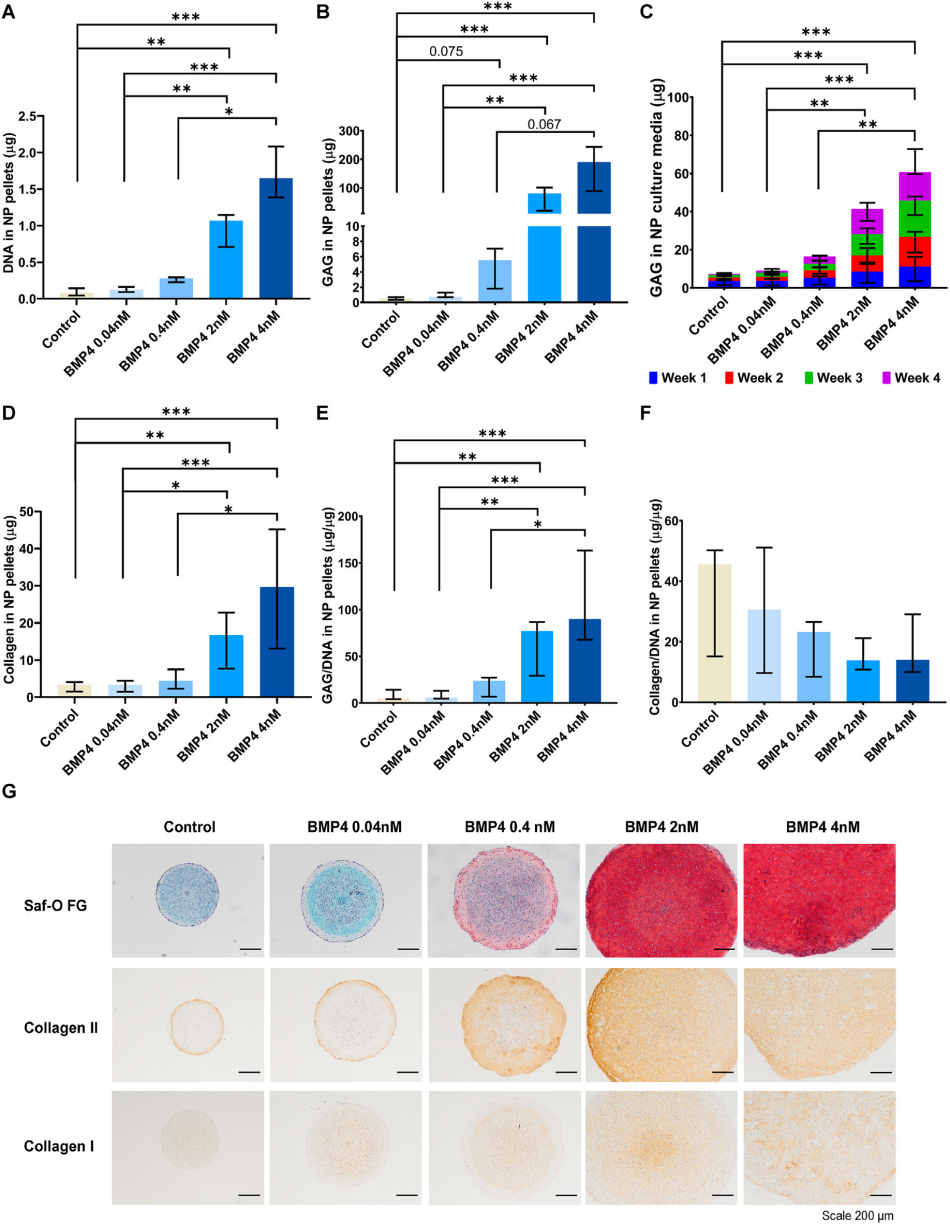
BMP-4 increased extracellular matrix production in NP cell pellet culture. Sheep nucleus pulposus (NP) cell pellets were cultured with (0.04 ​nM, 0.4 ​nM, 2 ​nM, 4 ​nM) or without bone morphogenetic protein-4 (BMP4) at four different concentrations for 28 days. The DNA (A), glycosaminoglycan (GAG) (B), collagen (D) content in pellets, and GAG release (C) in culture media were measured. E, F, The GAG and collagen content in pellets were normalized to DNA. Additionally (G) the ECM deposition in pellets was evaluated by Safranin-O Fast Green (Saf-O FG) staining for proteoglycan (red) and total collagen (cyan), and by immunohistochemistry staining for collagen type II and I (brown). Kruskal Wallis with the post-hoc test was used to determine differences among more groups. Median with interquartile range, 3 donors in triplicates, n ​= ​9, ∗P ​< ​0.05, ∗∗P ​< ​0.01, ∗∗∗P ​< ​0.001.

**Fig. 2:**
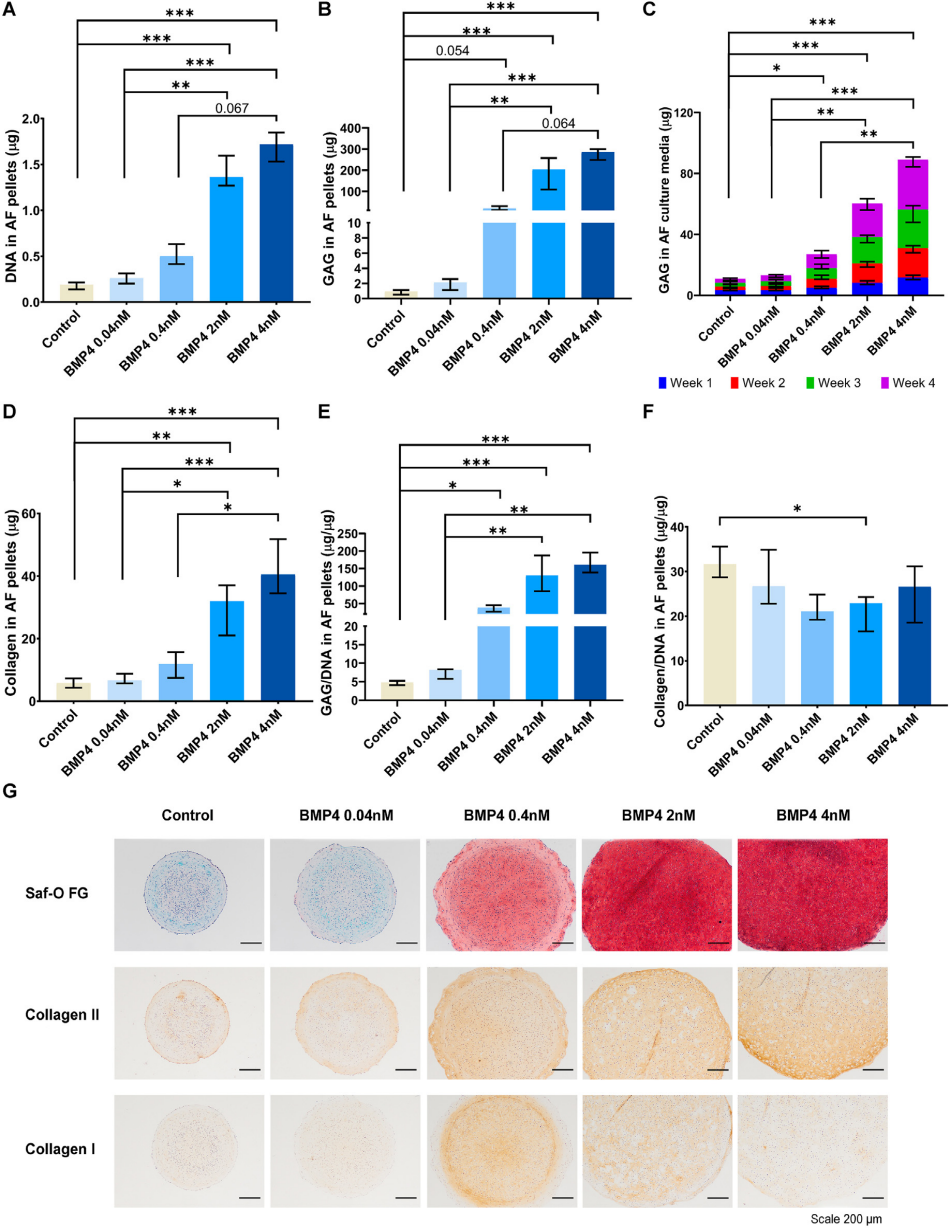
BMP-4 increased extracellular matrix production in AF cell pellet culture. Sheep annulus fibrosus (AF) cell pellets were cultured with (0.04 ​nM, 0.4 ​nM, 2 ​nM, 4 ​nM) or without bone morphogenetic protein-4 (BMP4) at four different concentrations for 28 days. The DNA (A), glycosaminoglycan (GAG) (B), collagen (D) content in pellets, and GAG release (C) in culture media were measured. E, F, The GAG and collagen content in pellets were normalized to DNA. Additionally (G) the extracellular matrix deposition in pellets were evaluated by Safranin-O Fast Green (Saf-O FG) staining for proteoglycan (red) and total collagen (cyan), and by immunohistochemistry staining for collagen type II and I (brown). Kruskal Wallis with the post-hoc test was used to determine differences among groups. Median with interquartile range, 3 donors in triplicates, n ​= ​9, ∗P ​< ​0.05, ∗∗P ​< ​0.01, ∗∗∗P ​< ​0.001.

During culture, the diameter of the pellets decreased over time in pellets cultured without or with a low dose of BMP-4 (0.04 ​nM) in both NP and AF cells (Supplementary Fig. 1), whereas pellets cultured with 0.4 ​nM BMP-4 maintained the same diameter throughout the culture period. At a concentration of 4 ​nM BMP-4 in NP cells (P ​< ​0.01 ​at 28 days) and 2 and 4 ​nM in AF cells (P ​< ​0.001 ​at 28 days), pellet size increased over time. As seen in Fig. 1, Fig. 2A, the DNA content was significantly higher in BMP-4 treated pellets, at doses higher than 2 ​nM, compared to non-treated controls in both NP (P ​< ​0.01 ​at 2 ​nM, P ​< ​0.001 ​at 4 ​nM) and AF (P ​< ​0.001 ​at 2 ​nM and 4 ​nM) cells. At day 1, the LDH activity was not different between BMP-4-treated and non-treated cells for both NP and AF cells (Supplementary Fig. 2). Normalized to DNA content in pellets at day 26, the relative LDH activity was decreased by BMP-4 at concentrations >0.4 ​nM in NP cells (P ​< ​0.01 ​at 0.4 ​nM, P ​< ​0.001 ​at 2 ​nM and 4 ​nM) and at ​> ​2 ​nM in AF cells (P ​< ​0.001 ​at 2 ​nM and 4 ​nM) compared to the non-treated control.

#### 3.1.2 BMP-4 increased ECM production in NP and AF pellet cultures

As shown in Fig. 1, Fig. 2B, the GAG content was increased by BMP-4 supplementation at concentrations of 2 ​nM or higher in both NP (P ​< ​0.001) and AF pellets (P ​< ​0.001). GAG content per DNA followed the same trend as GAG content (P ​< ​0.01 ​at 2 ​nM, P ​< ​0.001 ​at 4 ​nM in NP; P ​< ​0.001 ​at 2 and 4 ​nM in AF) (Fig. 1, Fig. 2E), as well as total GAG release in culture media (P ​< ​0.001 in NP and AF) (Fig. 1, Fig. 2C). Additionally, Safranin-O/Fast Green staining showed that in both NP and AF pellets, more proteoglycans were deposited (red) when treated with 0.4 ​nM or higher doses of BMP-4 (Fig. 1, Fig. 2G).

Similar effects were observed regarding the total collagen deposition in NP and AF pellets. At doses of 2 ​nM or higher, the total collagen content, although not collagen per DNA, was significantly increased compared to non-treated controls (P ​< ​0.01 ​at 2 ​nM, P ​< ​0.001 ​at 4 ​nM in NP and AF) (Fig. 1, Fig. 2F). Collagen type II immunopositivity was enhanced in pellets treated with 0.4 ​nM or higher dose of BMP-4 compared to non-treated controls (Fig. 1, Fig. 2G). While collagen type I deposition was not altered in BMP-4-treated NP pellets, it appeared to be increased in AF pellets cultured with 0.4 and 2 ​nM BMP-4 (Fig. 1, Fig. 2G).

#### 3.1.3 BMP-4 up-regulated the expression of cell proliferation marker, Ki-67, and ECM production-related gene, SOX-9

**Fig. 3:**
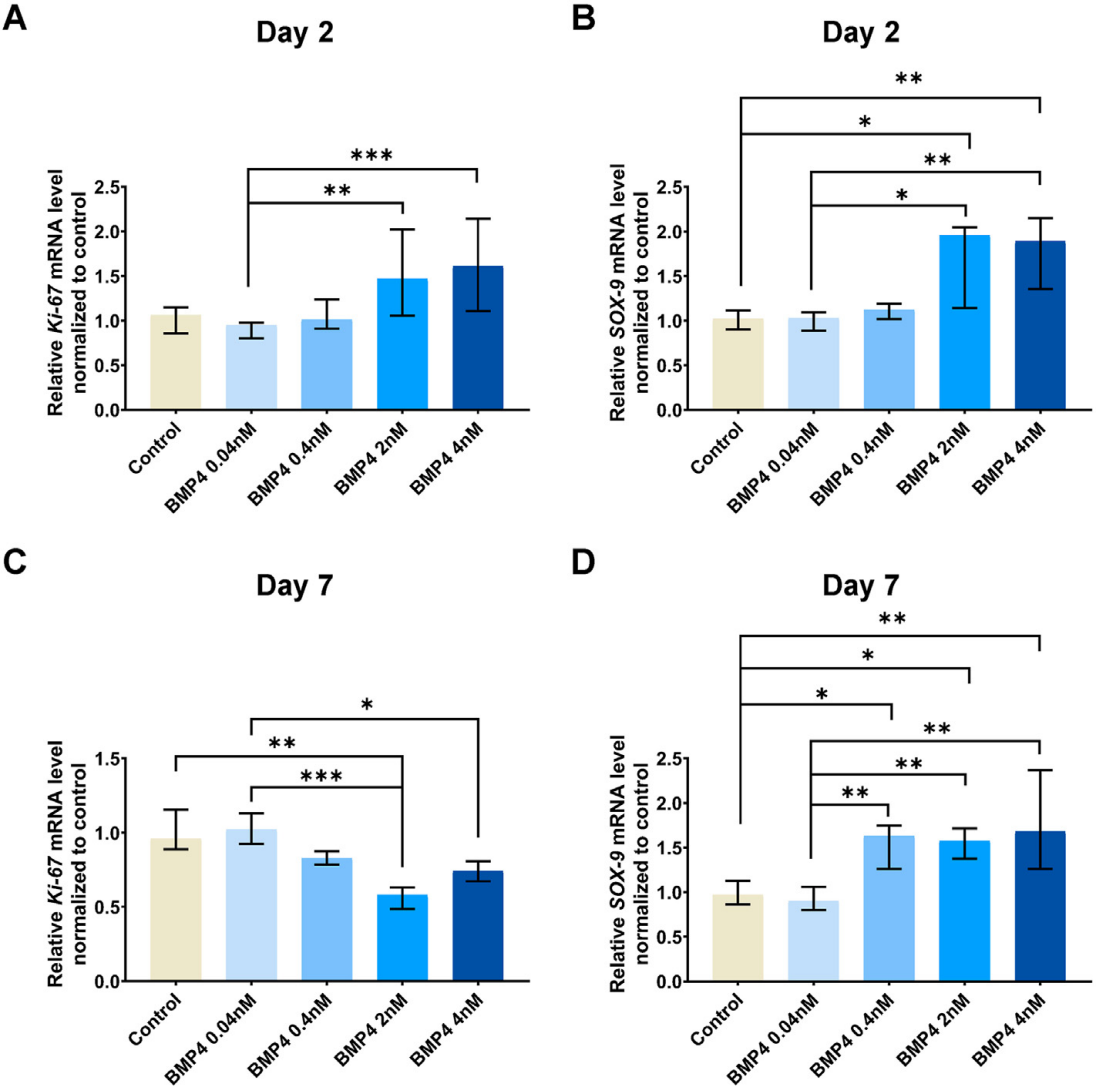
BMP-4 up-regulated the expression of cell proliferation marker, Ki-67, and ECM production-related gene, SOX-9. Sheep nucleus pulposus (NP) pellets were cultured with (0.04 ​nM, 0.4 ​nM, 2 ​nM, 4 ​nM) or without bone morphogenetic protein-4 (BMP-4) at four different concentrations for 2 and 7 days. The mRNA level of cell proliferation marker, nuclear Ki-67 protein (Ki-67) (A, C), and ECM production-related gene, SRY-box transcription factor-9 (SOX-9) (B, D) was measured by quantitive PCR at day 2 and day 7 respectively. Results were normalized to the control group. Kruskal Wallis with the post-hoc test was used to determine differences among groups. Median with interquartile range, 3 donors in triplicates, n ​= ​9, ∗P ​< ​0.05, ∗∗P ​< ​0.01, ∗∗∗P ​< ​0.001.

To further explore underlying molecular mechanism of BMP-4 induced cell proliferation and ECM production, BMP-4 treated pellets were collected to measure the expression of cell proliferation marker genes Ki-67, and ECM production related gene SOX-9 at day 2 and day 7 (Fig. 3). Ki-67 was significantly upregulated by BMP-4 at 2 ​nM ​(P ​< ​0.01) and 4 ​nM ​(P ​< ​0.001) compared to 0.04 ​nM at day 2 (Fig. 3A). However, it was downregulated at day 7 in which pelltes treated by BMP-4 at 2 ​nM ​(P ​< ​0.001 vs 0.04 ​nM, P ​< ​0.01 vs control) and 4 ​nM ​(P ​< ​0.05 vs 0.04 ​nM) compared to 0.04 ​nM and/or non-treated control (Fig. 3C). SOX-9 expression was significantly elavated by BMP-4 at 2 ​nM ​(P ​< ​0.05 day 2; P ​< ​0.05 vs control, P ​< ​0.01 vs 0.04 ​nM at day 7) and 4 ​nM ​(P ​< ​0.01 ​at day 2; P ​< ​0.05 vs control, P ​< ​0.01 vs 0.04 ​nM at day 7) compared to 0.04 ​nM and non-treated control at day 2 and day 7 (Fig. 3B and D). In addition, SOX-9 expression was elevated by BMP-4 at a lower concentration, 0.4 ​nM, at day 7 compared to non-treated control (P ​< ​0.05) and 0.04 ​nM ​(P ​< ​0.01) (Fig. 3D). These results are consistent with above foundings.

### 3.2 Effects of BMP-4 on disc regeneration in vivo

In order to evaluate the effects of BMP-4 on IVD regeneration in vivo, IVDD was induced by nuclectomy in sheep, followed by intradiscal injection of BMP-4. Animals recovered well from surgery. No side effects of injection were noted, and no significant weight loss was observed in any of the 4 sheep (Supplementary Table 2). One BMP-4 injected IVD was excluded from the analysis because BMP-4 had been injected in the AF rather than the NP.

#### 3.2.1 BMP-4 induced extradiscal bone formation, loss of subchondral bone, and cartilage ingrowth into the vertebral bone

**Fig. 4:**
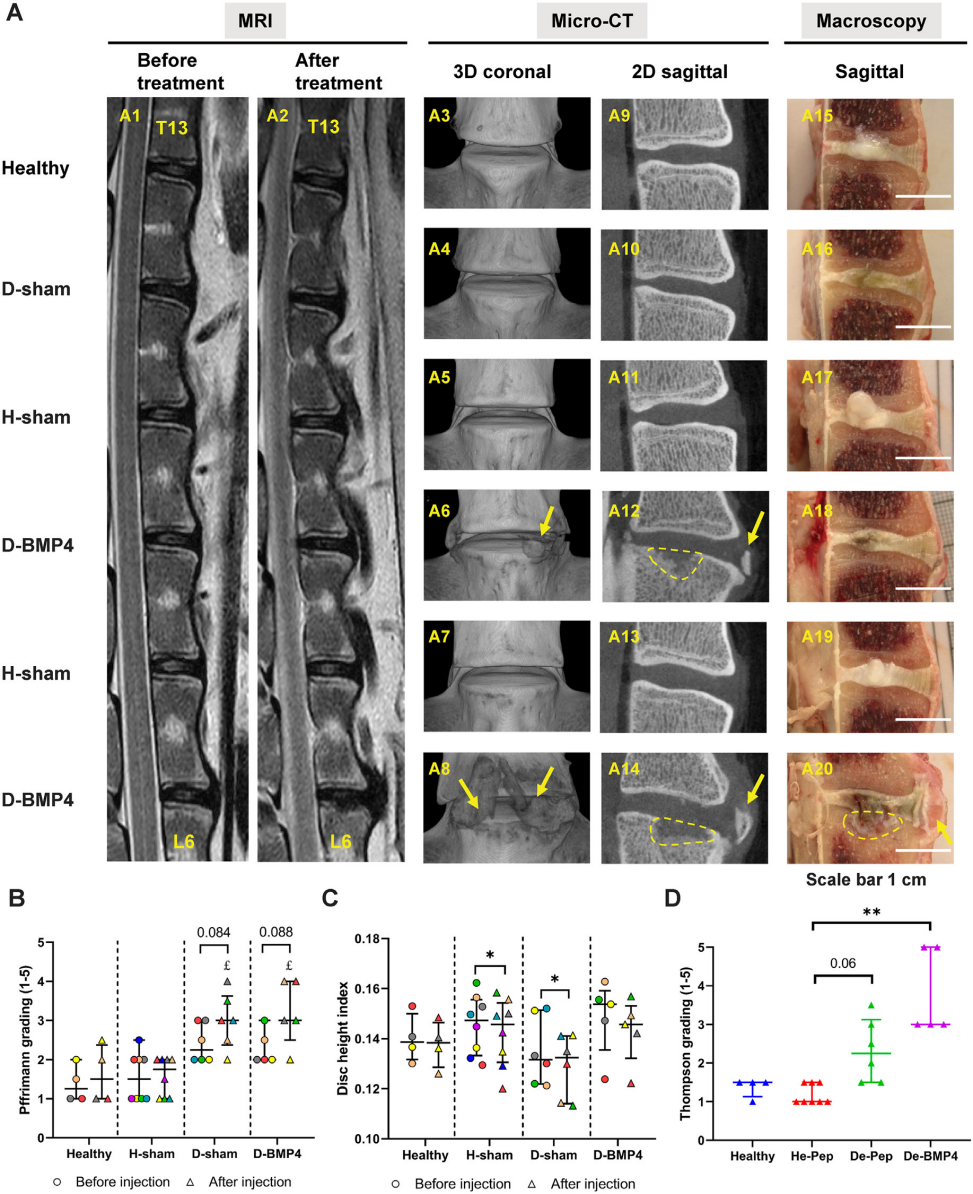
BMP-4 did not induce intervertebral disc regeneration but induced extradiscal new bone formation and subchondral bone remodeling. (A) Sheep lumbar intervertebral discs were measured by magnetic resonance image (MRI) before and three months after treatment (healthy discs (Healthy), healthy disc with random peptide injection (H-sham), degenerated discs with random peptide (D-sham) and BMP-4 injection (D-BMP4)), sagittal T2-weighted images (A1, before treatment, A2, after treatment), then IVDs were scanned by micro-CT, three dimensional (3D) coronal images (A 3–8) and two dimensional (2D) sagittal images (A 9–14), and discs were cut into two parts at middle sagittal (A 15–20). Arrow: new bone formation, circle: subchondral bone rupture. T2-weighted images were used to evaluate Pffrimann grading (B) and disc height index (C) before (circle) and after (triangle) treatment (the same color indicates the same disc within groups). 2D sagittal macroscopic images were used to evaluate Thompson grading (D). Kruskal Wallis with the post-hoc test was used to determine differences among groups in Pffrimann grading and disc height index before or after treatment separately and Thompson grading, using paired t-test to define the difference between before and after treatment in Pffrimann grading and disc height index. Median with interquartile range, sample size as independent dots, £ p ​< ​0.05, Paffrimann score of De-Pep and De-BMP4 vs He-Pep, ∗P ​< ​0.05, ∗∗P ​< ​0.01. Image A20 was reproduced from Lee N.N. et al. JOR Spine 4(2) (2021) e1162.

Before injection, IVDD was confirmed by a higher Pfirrmann grade in nuclectomy discs compared to control discs, although no difference in the DHI was noted (Supplementary Fig. 3). Three months after injection, Pfirrmann grade was similar between healthy and healthy sham-injected discs, while it was increased in degenerated sham-injected (P ​< ​0.05) and BMP-4-injected (P ​< ​0.05) discs when compared to healthy sham-injected discs. Pfirrmann grade in the degenerated disc at 3 months follow up was not significantly different from that observed before injection (Fig. 4B). As Fig. 4C shows, no difference in DHI was observed between the 4 conditions at 3 months after injection. DHI was significantly decreased 3 months after injection in sham-injected healthy (P ​< ​0.05) and degenerated (P ​< ​0.05) discs, but not in the healthy and BMP-4-injected degenerated discs. Upon macroscopic investigation, extradiscal bone formation and aberrant cartilage-like tissue ingrowth beyond the endplate (white tissue) were observed in BMP-4-injected discs (Fig. 4 A20). The Thompson score was not different between healthy and healthy sham-injected IVDs. It was significantly higher in BMP4-treated degenerated IVDs (P ​< ​0.01), but not in untreated degenerated discs (P ​= ​0.06), compared to healthy sham-injected IVDs (Fig. 4D). Additionally, according to the results from micro-CT, extradiscal new bone formation (Fig. 4 A6, A8) and subchondral bone loss (Fig. 4 A12, A14) were only found in degenerated BMP-4-injected discs (5 out of 5 discs), and as such the frequency was significantly higher in BMP-4-injected than non-treated discs (P ​< ​0.01) (Supplementary Table 3).

#### 3.2.2 In BMP-4-treated discs, regeneration was absent based on histological and biochemical analysis

**Fig. 5:**
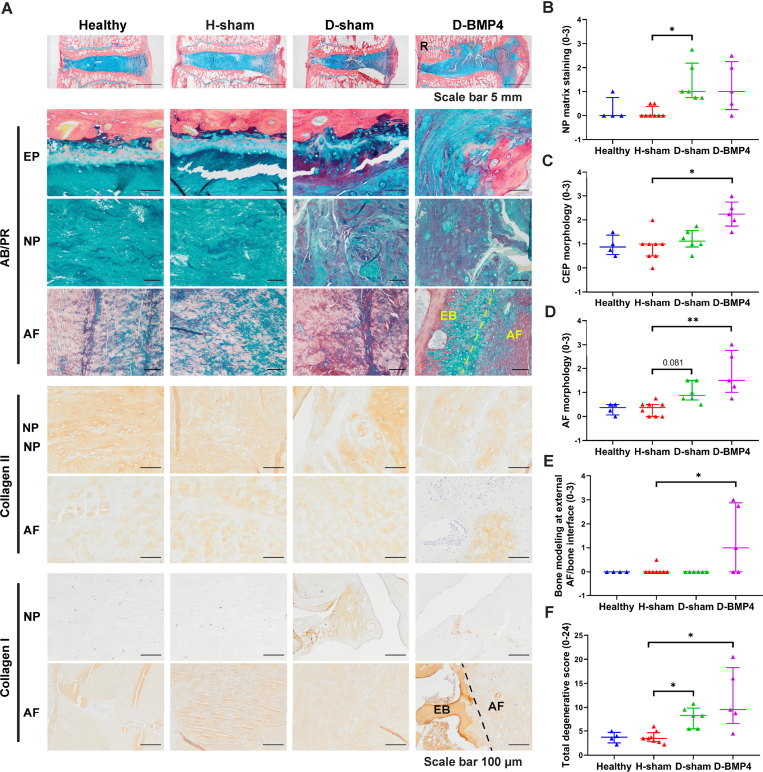
BMP-4 did not promote disc regeneration evaluated by histological degenerative grading. (A) Sheep IVDs were collected and performed histological staining including alcian blue and picrosirius red staining (AB/PR, proteoglycan (blue), collagen (red)) and immunohistochemical staining for collagen type I and II (healthy discs (Healthy), healthy disc with random peptide injection (H-sham), degenerated discs with random peptide (D-sham) and BMP-4 injection (D-BMP4)). Histological grading of disc degeneration was performed based on AB/PR, hematoxylin and eosin (H&E) and Safranin-O Fast Green (Saf O/FG) staining (H&E and Saf O/FG see Supplementary data 4). Endplate (EP), Annulus Fibrosus (AF), Nucleus Pulposus (NP), Extradiscal Bone (EB). Histological grading for NP matrix staining (B), cartilage endplate (CEP) morphology (C), AF morphology (D), bone modeling at the external AF/bone interface (E), and the total score of histological grading (F). The rest of grading parameters are in supplementary data 5. Kruskal Wallis with the post-hoc test was used to determine differences among groups. Median with interquartile range, sample size as independent dots, ∗p ​< ​0.05, ∗∗p ​< ​0.01. R: image of D-BMP-4 AB/PR staining macroscopy was reproduced from Lee N.N. et al. JOR Spine 4(2) (2021) e1162.

**Fig. 6:**
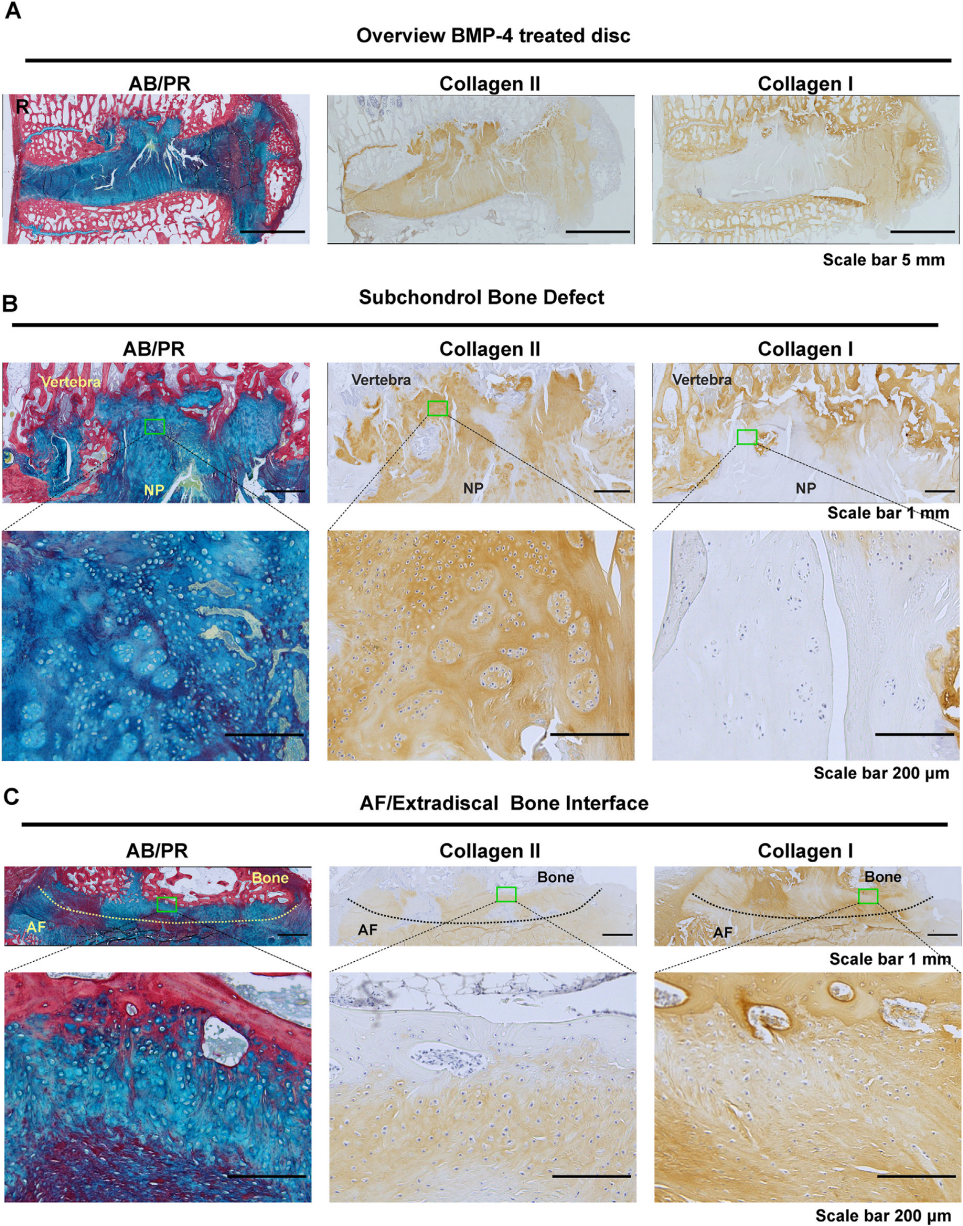
The overview and the area of subchondral bone defect and extradiscal bone in BMP-4 treated IVD. The overview (A) and the area of subchondral bone defect (B) and extradiscal bone (C) in BMP-4 treated IVD. Section stained by Alcian blue and picrosirius red staining (AB/PR, proteoglycan (blue), collagen (red)) and immunohistochemical staining for collagen type II and I (brown). R: image of D-BMP-4 AB/PR staining was reproduced from Lee N.N. et al. JOR Spine 4(2) (2021) e1162.

To further confirm the aforementioned results, histological and biochemical analyses were performed. As seen in Fig. 5A, AB/PR staining showed an irregular thickening of the EP in sham-injected degenerated IVDs, but not in healthy and healthy sham-injected IVDs. In BMP-4-injected degenerated IVDs, there was subchondral bone plate disruption with aberrant cartilaginous tissue. In the NP, heterogeneity, a decrease in Alcian blue and an increase in Picrosirius red staining were apparent in sham injected and BMP-4-injected degenerated IVDs, compared to healthy IVDs. In the AF, extradiscal bone formation and loss of lamellar structure were observed upon BMP-4 treatment. The degeneration score was performed based on AB/PR, H&E, and Saf-O fast green staining (H&E and Saf-O FG staining see Supplementary Fig. 4). Accordingly, the degeneration score of the NP matrix staining was significantly higher in sham-injected degenerated IVDs (P ​< ​0.05) compared to healthy sham-injected IVDs, but not in BMP4-treated degenerated IVDs (Fig. 5B), while degenerative grading on EP morphology (P ​< ​0.05), AF morphology (P ​< ​0.01), and bone modeling at the external AF/bone interface (P ​< ​0.05) was significantly higher with BMP-4 treatment when compared to sham-injected healthy IVDs, but not sham-injected degenerated IVDs (Fig. 5C–E). The total degeneration score was significantly higher in both sham-injected (P ​< ​0.05) and BMP-4-injected degenerated IVDs (P ​< ​0.05) compared to sham-injected healthy IVDs, but there was no difference between healthy and healthy sham-injected discs (Fig. 5F), nor between degenerated controls and BMP4-injected IVDs were observed. In the remaining 4 parameters of the degeneration score, including NP cell clustering, NP cell loss, tears and clefts, and demarcation between AF and NP, no significant differences were observed between treatments (Supplementary Fig. 5). Immunohistochemical staining of collagen type II and I is illustrated in Fig. 5A. In the NP, heterogeneity and loss of collagen type II was found in sham-injected and BMP-4-injected degenerated discs compared to healthy discs, but no difference was observed between BMP-4 treated and non-treated degenerated discs. All AF tissues were slightly collagen type II positive. Collagen type I deposition was apparent in the NP in sham-injected degenerated IVDs and also in BMP-4-injected degenerated IVDs. No difference was found regarding collagen type I staining in AF among treatments. Interestingly, when looking into the area of subchondral bone disruption in BMP-4 treated IVD (Fig. 6), subchondral bone was replaced with cartilage-like tissue, which was Alcian blue- and collagen II-positive, but negative for collagen type I. In these particular areas a high cell density and many chondrocyte-like cell clusters were observed. In the area of extradiscal bone formation, chondrocyte-like cells were surrounded by positive Alcian blue and slight collagen type II and I staining in the interface between AF and extradiscal bone.

**Fig. 7:**
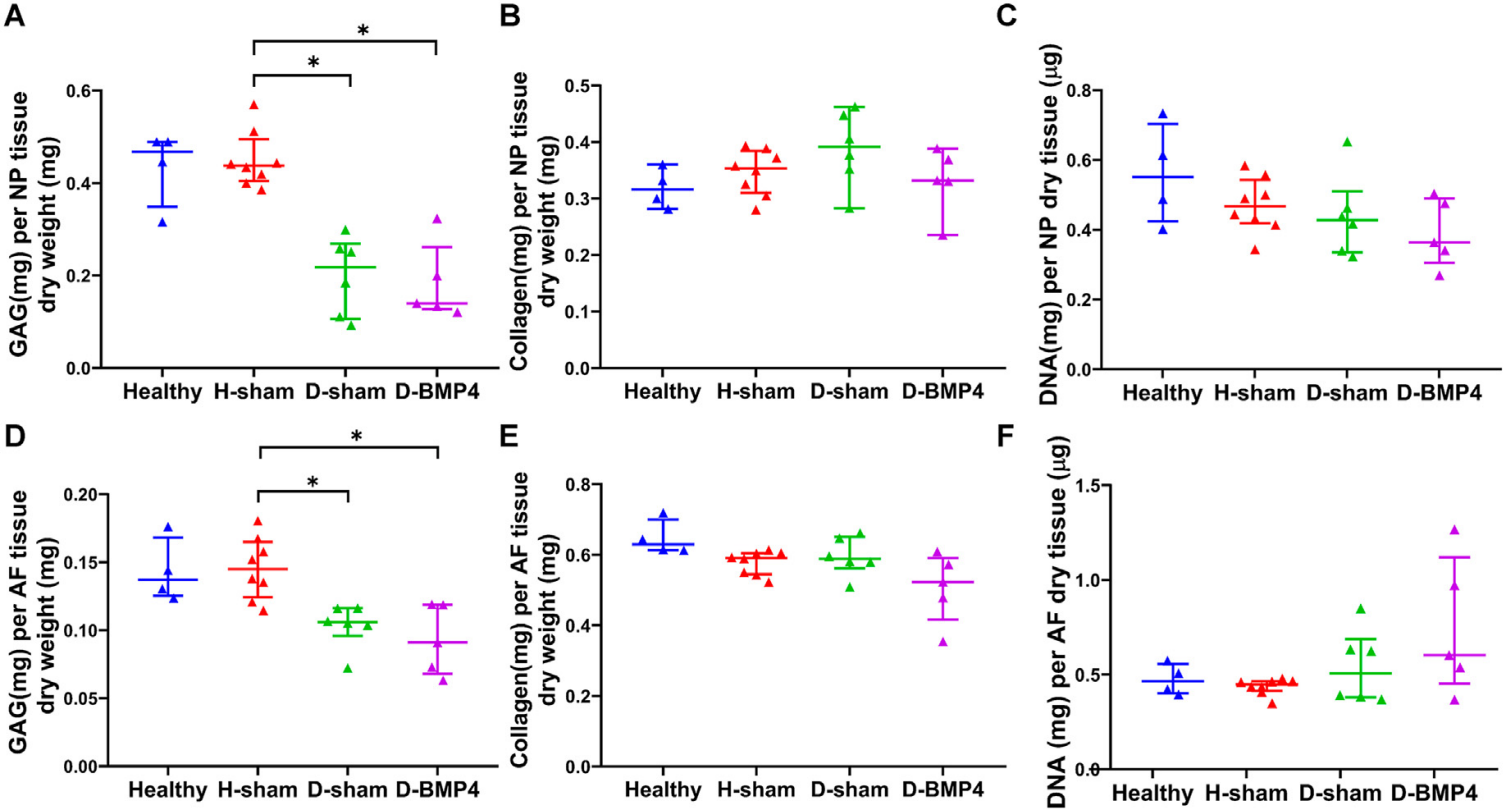
BMP-4 did not show positive effects on disc regeneration evaluated by biochemical analysis. Half part of the IVDs was collected for biochemical analysis. The glycosaminoglycan (GAG) (A, D), total collagen (B, E), and DNA (C, F) content were measured in both nucleus pulposus (NP) (A, B, C) and annulus fibrosus (AF) (D, E, F) tissue. GAG and total collagen content were normalized to tissue dry weight, and DNA content showed by μg per mg of tissue dry weight. Kruskal Wallis with the post-hoc test was used to determine differences among groups. Median with interquartile range, sample size as independent dots, ∗p ​< ​0.05.

As seen in Fig. 7A and D, the GAG content was reduced in sham-injected and BMP-4-injected degenerated IVDs compared to healthy sham-injected IVDs in both NP and AF (P ​< ​0.05), and no difference was observed between healthy and healthy sham-injected IVDs. No difference was observed in collagen and DNA content in NP and AF (Fig. 7B, C, E, F).

### 3.3 Osteoclast numbers in the subchondral bone

**Fig. 8:**
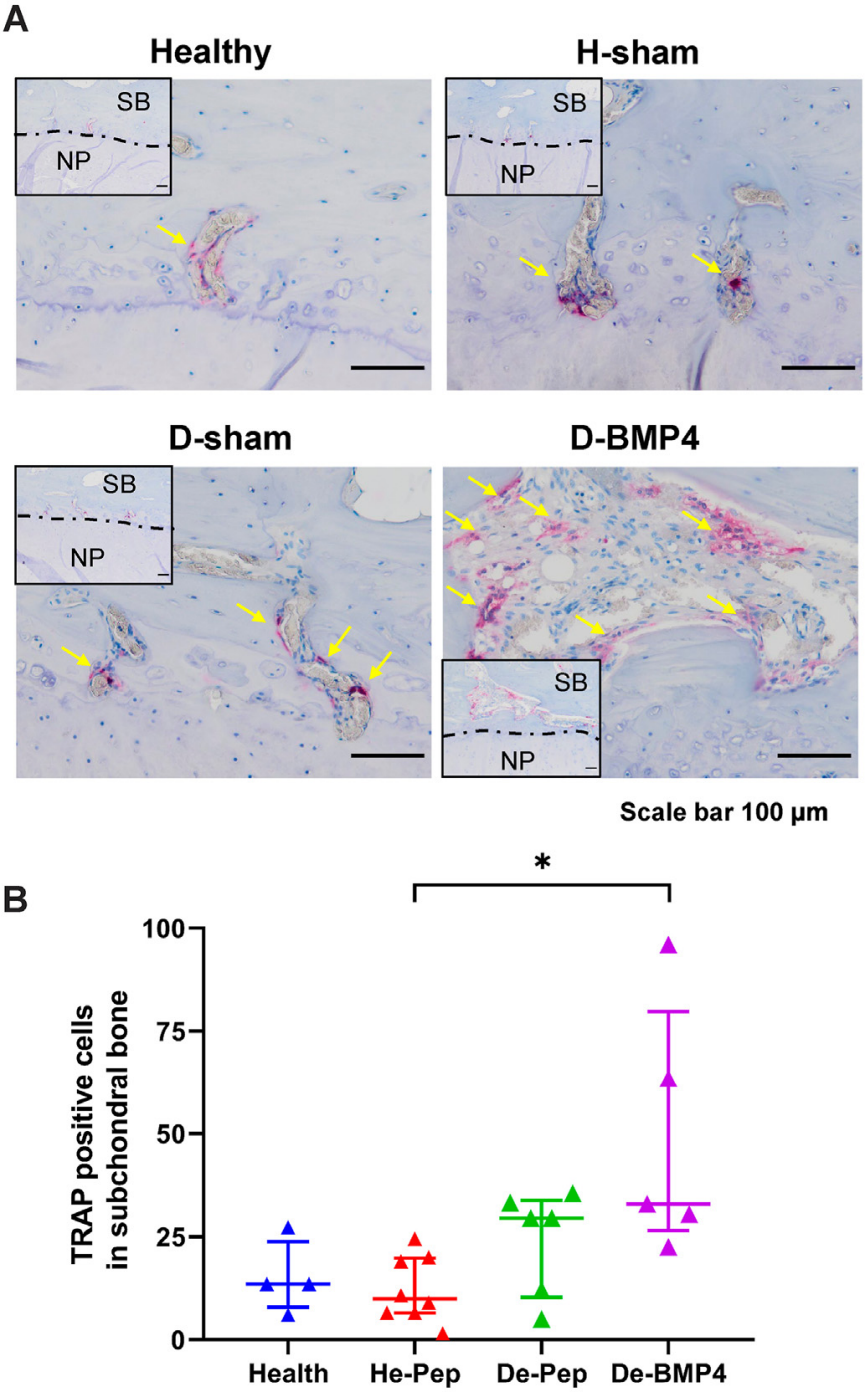
Osteoclast numbers were increased in subchondral bone of discs that were treated with BMP-4 and had subchondral bone rupture. (A) Tartrate-resistant acid phosphatase (TRAP) staining was performed to detect osteoclast in subchondral bone of IVDs (healthy discs (Healthy), healthy disc with random peptide injection (H-sham), degenerated discs with random peptide (D-sham) and BMP-4 injection (D-BMP4)). TRAP positive cells (yellow arrow) were counted in both sides of subchondral bone (B). Corner images at low magnification show the position was located at the subchondral bone, SB: subchondral bone, NP: nucleus pulposus. Kruskal Wallis with the post-hoc test was used to determine differences among groups. Median with interquartile range, sample size as independent dots, ∗p ​< ​0.05.

To check whether the subchondral bone loss in the BMP-4 treated IVDs was related to osteoclast activity, TRAP staining was used to detect osteoclasts. As Fig. 8A shows, osteoclasts were found in IVDs treated by BMP-4, especially in the area around subchondral bone loss. The number of TRAP positive multinucleated cells was significantly higher in the IVDs treated with BMP-4 (P ​< ​0.05), but not sham-injected degenerated IVDs when compared to sham-injected healthy IVDs (Fig. 8B).

## 4 Discussion

IVD regeneration has been proposed as a promising strategy to treat discogenic CLBP. Inducing regeneration by growth factors has been widely investigated. In this study, we report that although BMP-4 showed powerful regenerative effects in vitro by promoting cell proliferation and ECM anabolism in sheep NP and AF cells, extradiscal bone formation and aberrant cartilage tissue overgrowth associated with subchondral bone loss were found when BMP-4 was applied intradiscally in vivo.

In NP pellet culture, cell proliferation and deposition of proteoglycan and collagen type II, but not collagen type I, was dose-dependently increased by BMP-4. These findings are consistent with previous studies, where BMP-4 was used to treat human NP cells or overexpressed in bovine NP cells using adenoviruses [23,31]. In addition, in the current study, the effect of BMP-4 in promoting cell proliferation was confirmed by increasing Ki-67 expression by the high concentration of BMP-4 on day 2, while decreasing Ki-67 expression on day 7 may be due to inverse feedback. Moreover, BMP-4 up-regulated SOX-9, indicating that BMP-4 enhanced ECM deposition may be via SOX-9. In the current study, AF cells treated with BMP-4 showed similar effects as NP cells, including enhanced production of proteoglycans and collagen II and increased proliferation. However, in contrast to NP cells, the production of collagen I appeared to be increased in AF at low concentrations BMP-4, then declined with further increasing BMP-4 concentration. The underlying mechanism may warrant further study to tune stimulation of ECM production by BMP-4 in AF cells. In AF tissue, the proteoglycan and collagen II content gradually decreases from the inner to outer AF, while collagen I increases [9]. Proteoglycan content in the inner AF may provide functional compensation when the proteoglycan declines in NP with disc degeneration [32]. Although the majority of studies on IVDD focused on NP tissue, pathological changes in AF tissue are commonly associated with pain and disability and therefore AF repair merits attention [33,34].

In contrast to our in vitro data, intradiscal injection of BMP-4 did not promote disc regeneration in vivo. Severe side effects were found in BMP-4-treated discs, including ectopic bone formation, subchondral and vertebral bone loss, and aberrant growth of cartilage-like tissue through the EP to the vertebral bone. The extradiscal bone formation could be induced by BMP-4 leakage and diffusion from the IVD. In the current study, the volumes injected were limited, as was needle size. A recent study showed that this risk of leakage could be minimized to zero by using small injection volume, small needle diameters [35]. Hence direct leakage through the injection tract is less likely, and extradiscal bone formation is probably due to diffusion. BMPs are known as potent inducers of bone formation. Overexpression of BMP-4 in rat adipose-derived stromal cells, NIH/3T3 and C2C12 ​cells induced osteogenic and/or endochondral bone formation after implanting these cells into immunodeficient mice [36,37]. Similarly, extradiscal bone formation also occurred in a previous study after intradiscal injection of BMP-7 at a similar dose in a canine spontaneous IVDD model, although no effects the endplate nor subchondral bone were observed [20].

Besides extradiscal bone formation, BMP-4 also induced subchondral and vertebral bone loss and aberrant cartilage-like tissue through the EP to the vertebral bone. This change is highly similar to a Schmorl's node, in which the soft disc tissue bulges out into the adjacent vertebrae through an EP defect [38]. The pathogenesis of Schmorl's nodes is still uncertain. In our case, it may be associated with weakened vertebral bone and/or cartilage EP. One explanation could be that the loss of vertebral bone initiated this Schmorl's node-like change. In the current study, although the osteoclast number along subchondral bone was not different between BMP-4-treated and non-treated degenerated discs, the osteoclast number was significantly higher in the area around the subchondral bone loss in IVDs treated with BMP-4, indicating BMP-4 may induce osteoclast proliferation or activation which causes bone resorption. Despite the increasing osteoclast numbers, it could be a consequence of EP rupture. Our hypothesis could be confirmed by a previous study that showed overexpression of BMP-4 in bone induced severe osteopenia with increasing osteoclast number and phosphorylated Smads 1/5/8 BMP signaling in mice [39]. This suggests that BMP-4 may be associated with both bone formation and bone resorption. These contradictory effects on bone metabolism were also reported for BMP-2 when it was used for transforaminal lumbar interbody fusion showing evidence of transient vertebral endplate osteoclastic activity in radiographs [40]. Studies suggested the BMP-2-induced osteolysis may be related to an increase of inflammation [40,41]. A recent study showed circulating BMP-4 levels were elevated during thoracic surgery and positively correlated with pro-inflammatory cytokines, including IL-1β and TNF-α, and suggested that BMP-4 may exert pro-inflammatory properties via cyclooxygenase-II signaling pathways [42]. Whether the BMP-4-related bone resorption in the current study is associated with inflammation remains unclear, and warrants further study. Another potential explanation should be that the deteriorated EP initiated Schmorl's node-like change. The effect of BMP-4 on EP has, however, rarely been studied. A previous study showed that injection of 100 ​μg BMP-2 in a rabbit IVDD model caused EP hypertrophy [43]. A better understanding of the effects and underlying mechanism of BMP-4 in bone and cartilage-like tissue will facilitate further evaluation of BMP-4 for IVD regeneration or bone regeneration for spine fusion.

Why the NP and AF cells did not respond to BMP-4 by enhancing matrix production in vivo, while other cells and in vitro cultures did, is not clear. One recent study showed that a combination of BMP-4, TGF-β_3_, and chitosan hydrogel promoted IVD regeneration in a rabbit model of acute disc injury [44]. However, the outcomes could be influenced by the animal model, additional growth factor, and hydrogel compared to the current study. Whether BMP-4 alone could promote disc regeneration in large animals still is unknown. One of the explanations may be that the BMP-4 has not been injected at enough dose or present sufficiently long to change NP cell behavior. However, the injected dose was around 500 times higher than the concentrations used in our in vitro model. Interestingly, a high density of chondrocyte-like cells and clusters were found in Schmorl's node-like change area, and these cells were surrounded by proteoglycan and collagen II rich matrix. This was not observed in one of our earlier studies where BMP-7 was injected, where only extradiscal bone formation was seen [20]. The observation that the endplate does respond to the injected BMP-4 also suggests the quantity injected was biologically active. According to the previous study decreasing the injected dose could ease the side effects but may not help to promote regeneration effects [20]. No such increase in proteoglycan-rich tissue was observed in the NP area. It is plausible that this relates to the closer proximity to the subchondral bone blood vessels and inherently an environment that has better access to nutrients and subjected to the subchondral bone signals. At the same time, this may also mean that the differential responsivity of the different tissues of the IVD may preclude the applications of such strategies. Extradiscal side effects may be overcome by gradual administration of growth factor. Weekly intradiscal injection of low doses of TGF-β_1_ for 4 weeks induced improvement in a mouse model of IVDD by increasing annular-derived chondrocytic cell migration into the NP, expressing aggrecan and collagen type II without side effects [45]. BMP-2 or BMP-2/7 heterodimer and a fibrin/hyaluronic acid hydrogel were combined to treat mild IVDD in a goat model [14]. This slow release system appeared to be safe, without signs of adverse effects, although no beneficial effect was observed in the treated discs, possibly due to the low dose of 1 ​μg BMP [14]. Using these slow release drug delivery technologies to control local concentration and prolong bioactivity of BMP-4 after intradiscal delivery may warrant further study. Ideally, this would be developed and optimized in ex vivo models. However, none of these are still capable of a complete recap of IVD degeneration, including proteoglycan loss and replacement by collagen I. In addition, the harsh microenvironment, limited nutrition, and inflammation associated with degeneration could be some of the other possible explanations for this ineffective BMP-4 treatment [46]. Low grade inflammation has been shown previously in the experimental nuclectomy canine IVD and annulus fibrosis injured sheep IVD [26,47]. The inflammation induced by possibly BMP-4 itself may further accelerate the degeneration instead of promoting regeneration. Overall, given the complete absence of any regenerative effect of the injected BMP-4 on NP or AF tissue in the current large animal model, achieving intradiscal regeneration in IVDs with established degeneration may provide a challenge. Possibly more factors like injection dose and method, limited nutrient, and inflammation should be taken into account when using growth factors for IVD regeneration.

## 5 Conclusion

In conclusion, BMP-4 promoted chondrogenic ECM production and cell proliferation of NP and AF cells in vitro. Intradiscal injection of a single dose of BMP-4 failed to halt disc degeneration or induce disc regeneration. Instead, extradiscal bone formation, endplate hypertrophy and Schmorl's node-like changes were induced. Therefore, a similar dose of soluble BMP-4 should not be considered for directly intradiscal injection as a strategy for IVD regeneration.

## Funding

This project has received funding from the European Union's Horizon 2020 research and innovation programme under Marie Sklodowska-Curie CoFund, grant agreement 801,540, under grant agreement no 825925 and the Dutch Arthritis Foundation (LLP12 and LLP22).

## Author contribution

Jie Du: Methodology, Investigation, Formal analysis, Writing - Original Draft, Writing - Review & Editing. João P. Garcia: Methodology, Investigation, Formal analysis, Writing - Review & Editing. Frances C. Bach: Formal analysis, Writing - Review & Editing. Anna R. Tellegen: Formal analysis, Writing - Review & Editing. Sibylle Grad: Methodology, Writing - Review & Editing. Zhen Li: Methodology, Writing - Review & Editing. René M Castelein: Methodology, Writing - Review & Editing. Björn P. Meij: Methodology, Investigation, Writing - Review & Editing. Marianna A. Tryfonidou: Conceptualization, Methodology, Writing - Review & Editing,. Laura B. Creemers: Conceptualization, Methodology, Writing - Review & Editing, Funding acquisition.

## Declaration of competing interest

The authors have no conflicts of interest relevant to this article.
