# Supplementary material for: Intradiscal injection of human recombinant BMP-4 does not reverse intervertebral disc degeneration induced by nuclectomy in sheep
Source: J Orthop Translat. 2022 Sep 23;37:23–36. doi: 10.1016/j.jot.2022.08.006 (PMC9513727; doi:10.1016/j.jot.2022.08.006)
Supplement: Multimedia component 1 [file mmc1.docx]

**
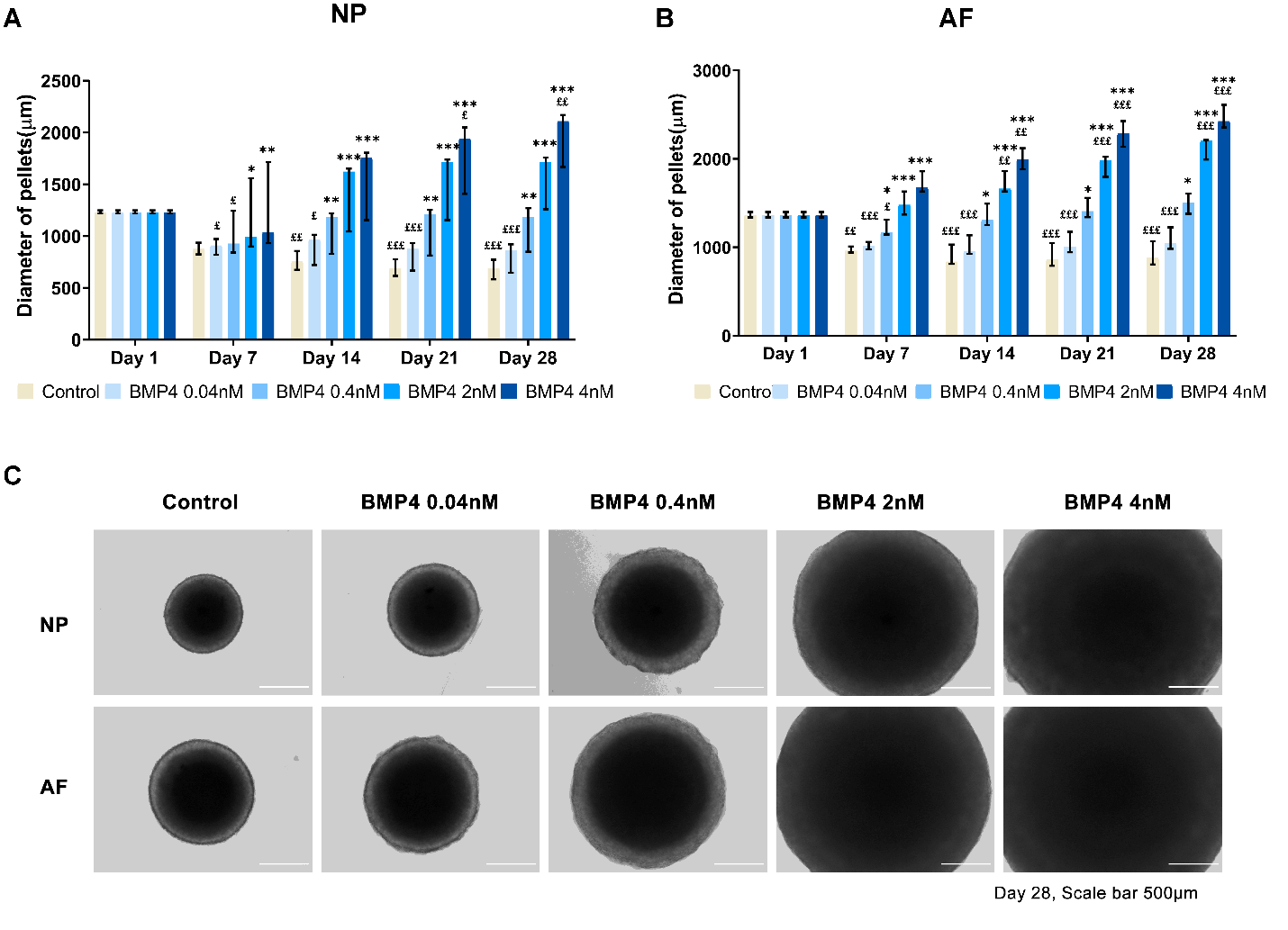
**

**Supplementary figure 1.** **BMP-4 increased pellet size over time and with increasing of dose.**

Sheep nucleus pulposus (NP) and annulus fibrosus (AF) cell pellets were cultured with (0.04 nM, 0.4 nM, 2 nM, 4 nM,) or without BMP-4 at four different concentrations for 4 weeks. The diameter of pellets of NP (**A**) and AF (**B**) was measured at day 1 and every week. **C,** The Pellet’s size of NP and AF after 4 weeks culture. Kruskal Wallis with the post-hoc test was used to determine differences among more groups. Median with interquartile range, 3 donors in triplicates, n=9, £ p< 0.05, ££ p< 0.01, £££ p< 0.001 vs day 1 within each condition by time points, * p< 0.05, ** p< 0.01, *** p< 0.001 vs control within each time point by conditions.

**
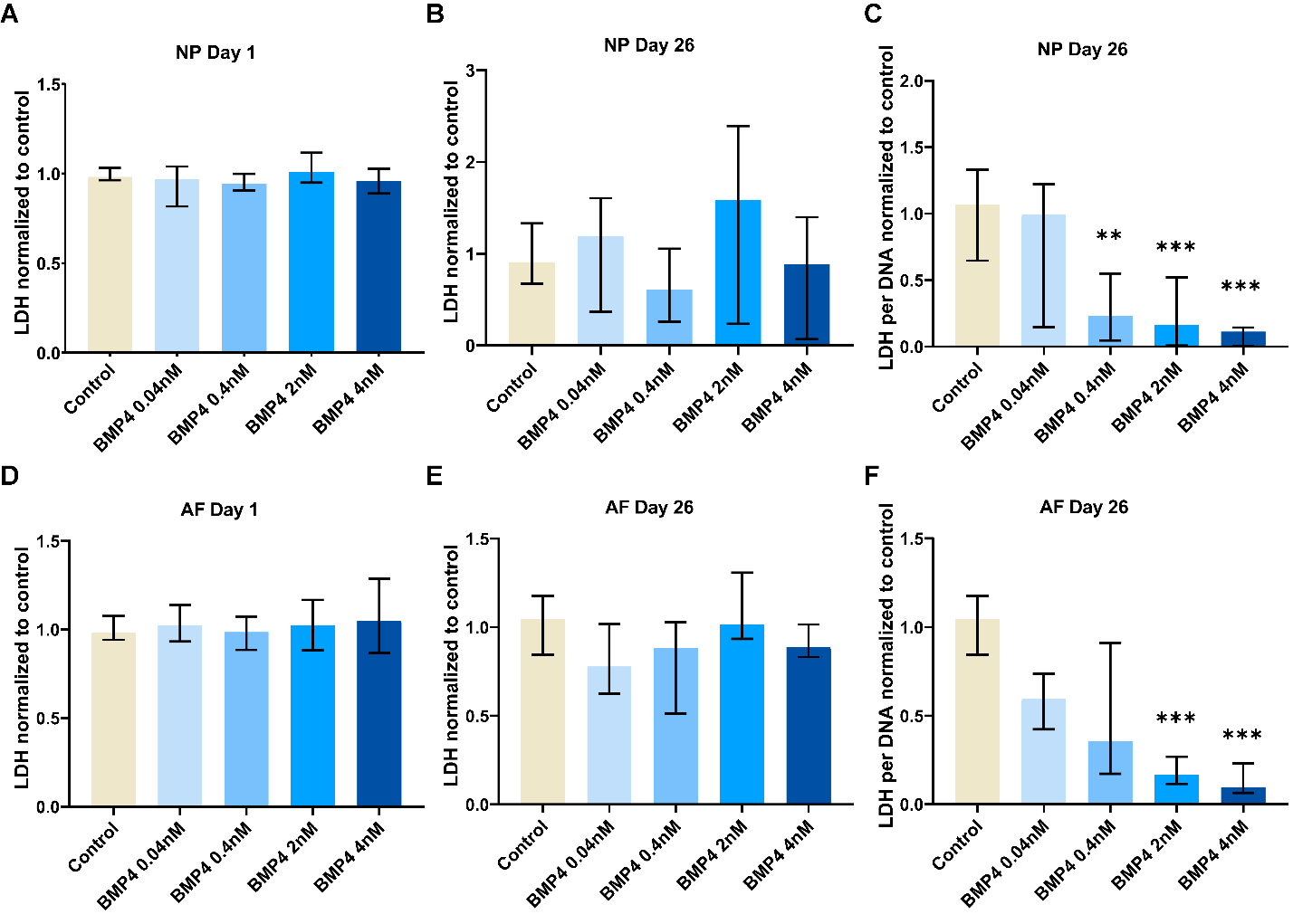
Supplementary figure 2.** **No cytotoxicity was found after treatment by BMP-4 in sheep NP and AF pellets.**

Sheep nucleus pulposus (NP) **(A, B, C)** and annulus fibrosus (AF) **(D, E, F)** cell pellets were cultured with (0.04 nM, 0. 4 nM, 2 nM, 4 nM,) or without BMP-4 at four different concentrations for 4 weeks. Cytotoxicity was measured by detection of lactate dehydrogenase (LDH) activity in culture media at day 1 (**A, D**) and day 26 (**B, E**) for 1 day incubation with BMP-4, and results were normalized to non-treated control. **(C, F)** LDH activity per DNA at day 26 were calculated and normalized to non-treated control. Cytotoxicity was measured using the Cytotoxicity Detection Kit (4744926001, Roche Diagnostics GmbH, Mannheim, Germany) according to the manufacturer’s protocol. Kruskal Wallis with the post-hoc test was used to determine differences among more groups. Median with interquartile range, 3 donors in triplicates, n=9, ** p< 0.01, *** p< 0.001 vs control.

**
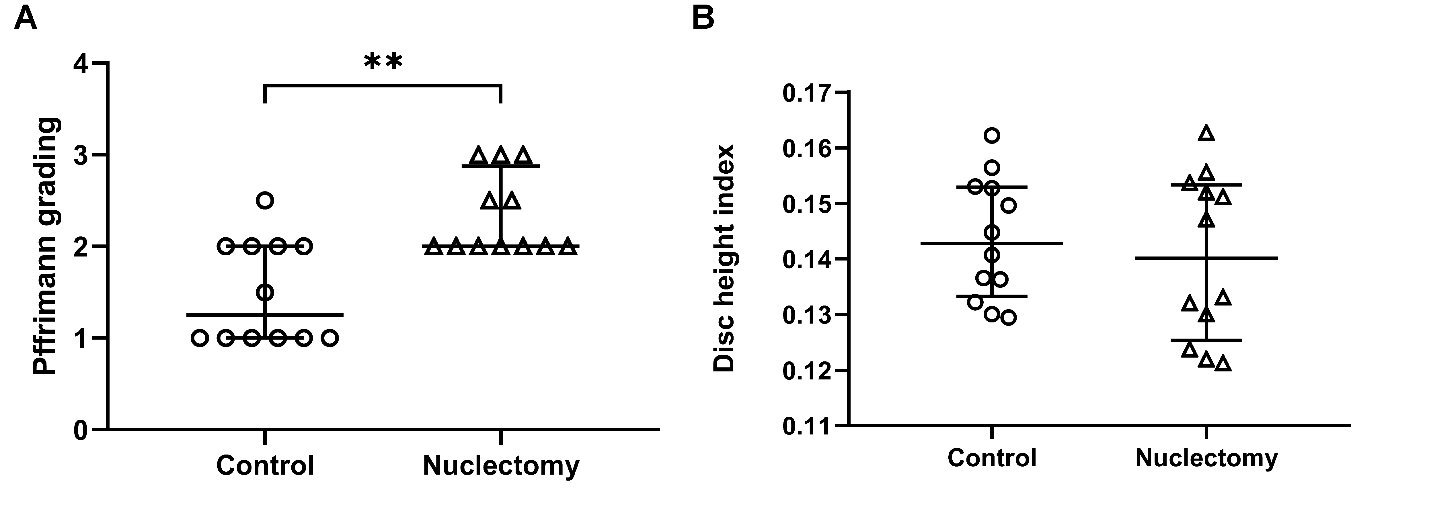
**

**Supplementary figure 3. IVD degeneration induced by nucleotomy.**

Three months after nucleotomy conduction, magnetic resonance image (MRI) was used to evaluate degeneration of disc, then Pffrimann grading (**A**) and disc height index (**B**) were performed. Statistical analysis performed using Mann-Whitney U test. Median with interquartile range, sample size as independent dots, ** p< 0.01.

**
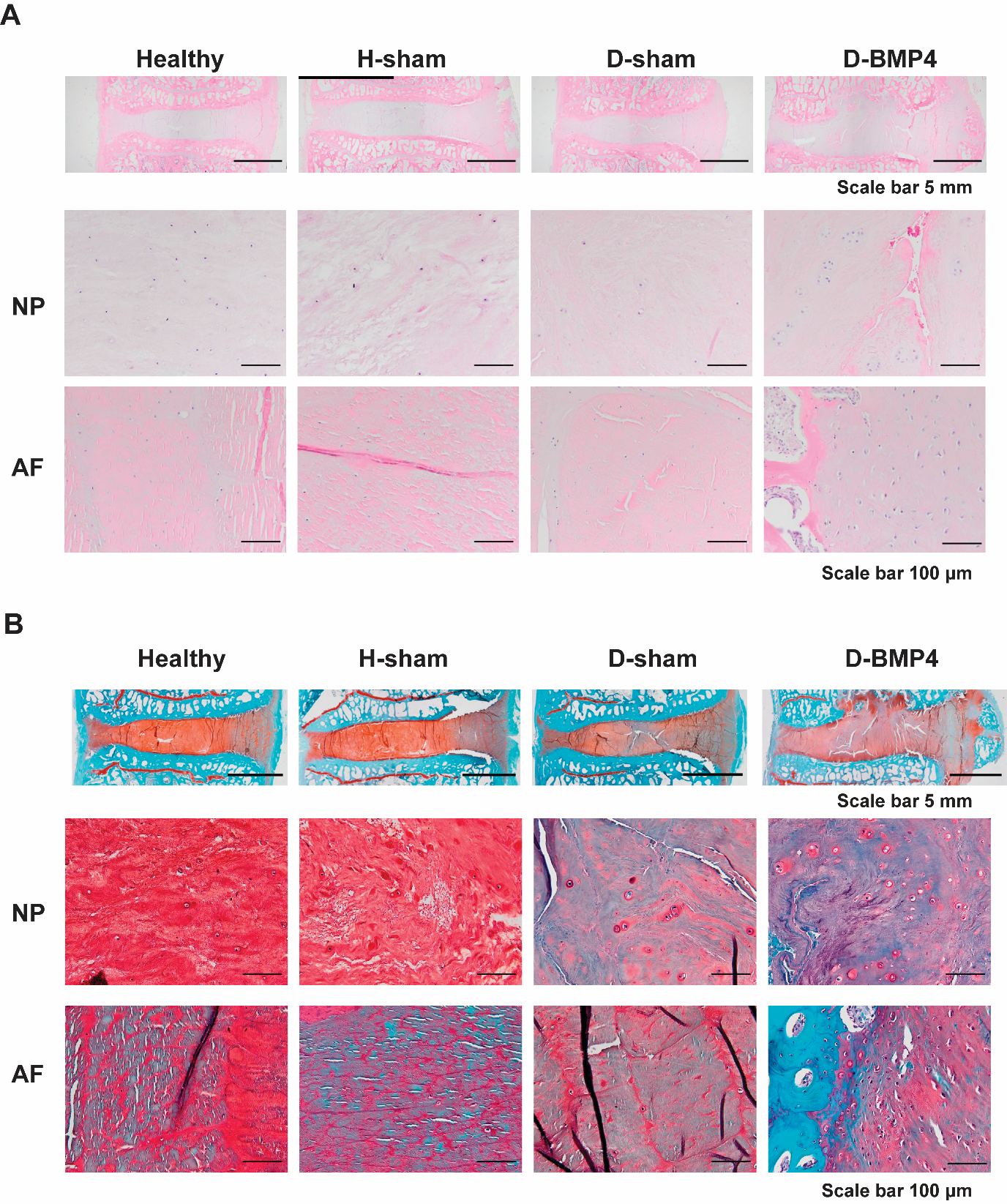
**

**Supplementary figure 4. BMP-4 did not show positive effects on disc regeneration evaluated by histological analysis.**

(**A**) hematoxylin and eosin (H&E) and (**B**) Safranin-O Fast Green (Saf O/FG) staining for the discs (healthy discs (Healthy), healthy disc with random peptide injection (H-sham), degenerated discs with random peptide (D-sham) and BMP-4 injection (D-BMP4)). Nucleus Pulposus (NP), Annulus Fibrosus (AF).


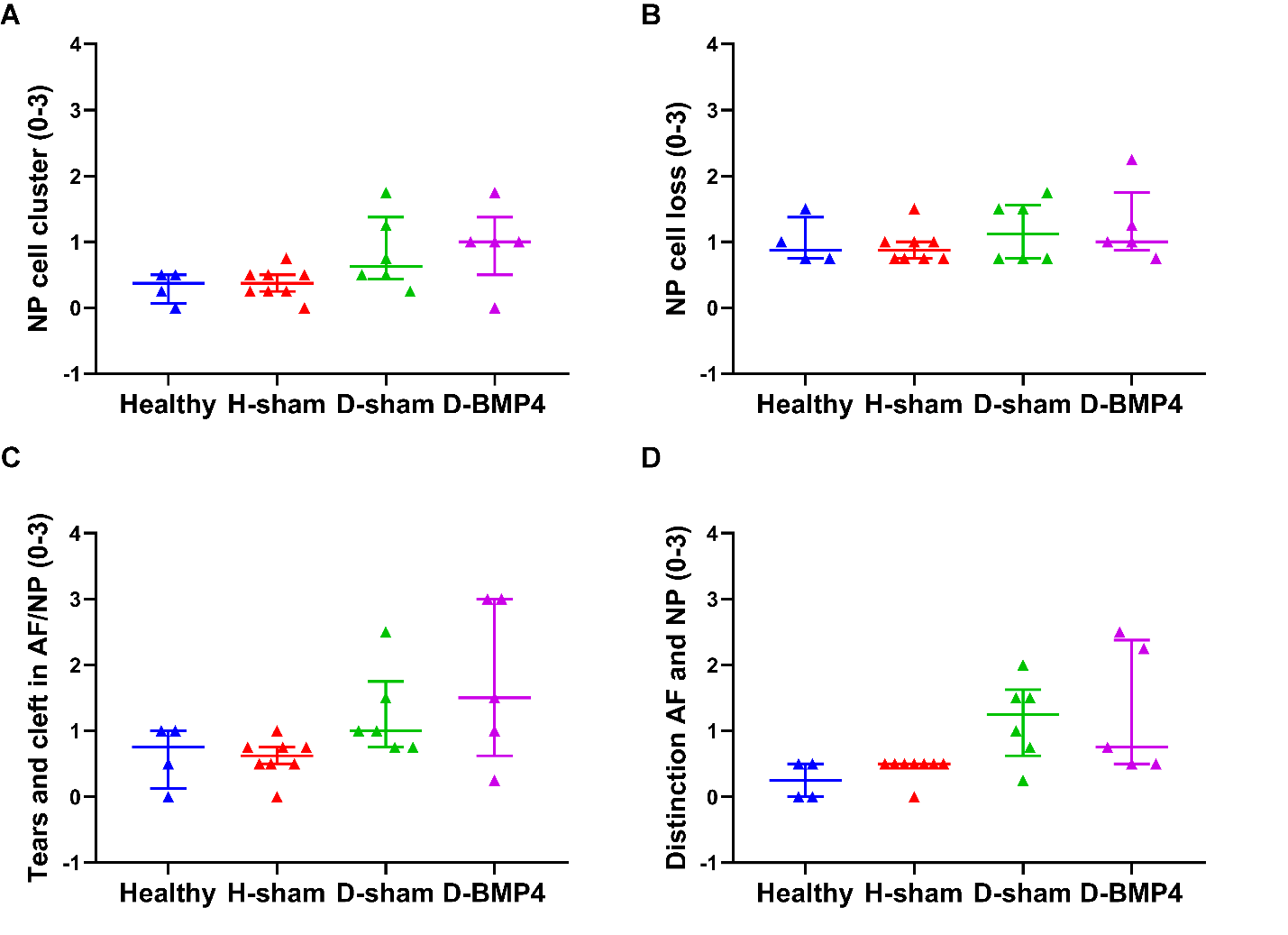


**Supplementary figure 5. BMP-4 did not show positive effects on disc regeneration evaluated by histological grading (parameters show no difference among groups).**

Histological grading of disc degeneration was performed based on alcian blue and picrosirius red (AB/PR), hematoxylin and eosin (H&E) and Safranin-O Fast Green (Saf O/FG). Histological grading for NP cell cluster **(A)**, NP cells loss (**B**), tears and cleft in AF/NP (**C**), distinction AF and NP (**D**). Kruskal Wallis with the post-hoc test was used to determine differences among groups. Median with interquartile range, sample size as independent dots.

**Supplementary Table 1. Sheep discs and treatment.**

|  | | **Treatment** | | | |
| --- | --- | --- | --- | --- | --- |
|  | | **Sheep 1** | **Sheep 2** | **Sheep 3** | **Sheep 4** |
| **IVDs** | **T13-L1** | Healthy | Healthy | Healthy | Healthy |
|  | **L1-2** | D-sham | D-BMP4 | D-BMP4 | D-sham |
|  | **L2-3** | H-sham | H-sham | H-sham | H-sham |
|  | **L3-4** | D-BMP4 | D-sham | D-sham | D-BMP4* |
|  | **L4-5** | H-sham | H-sham | H-sham | H-sham |
|  | **L5-6** | D-BMP4 | D-sham | D-BMP4 | D-sham |

* This disc was excluded from results. Because BMP-4 was injected to AF.

**Supplementary Table 2. Sheep’s weight before and 3 months after treatment.**

|  |  | **Weight (kg)** | |
| --- | --- | --- | --- |
|  |  | **Before treatment** | **Three months after treatment** |
| **Animals** | **Sheep 1** | **97** | **95** |
|  | **Sheep 2** | **86** | **85** |
|  | **Sheep 3** | **78** | **81** |
|  | **Sheep 4** | **75** | **77.5** |

**Supplementary Table 3. Extradiscal new bone formation and subchondral bone rupture after treatment.**

|  | |  | **Extradiscal new bone formation** | | **Subchondral bone rupture** | |
| --- | --- | --- | --- | --- | --- | --- |
| **Treatment** |  | | **No** | **Yes** | **No** | **Yes** |
|  | **Healthy (n=4)** | | **4** | **0** | **4** | **0** |
|  | **H-sham (n=8)** | | **8** | **0** | **6** | **2** |
|  | **D-sham (n=6)** | | **6** | **0** | **6** | **0** |
|  | **D-BMP4 (n=5)** | | **0** | **5*** | **0** | **5*** |

* Frequency is significant different (*P*< 0.01) than De-Pep, tested by Fisher’s Exact Test.
